# Self-expandable metallic stenting as a bridge to surgery for malignant colorectal obstruction: pooled analysis of 426 patients from two prospective multicenter series

**DOI:** 10.1007/s00464-018-6324-8

**Published:** 2018-07-13

**Authors:** Masafumi Tomita, Shuji Saito, Shinichiro Makimoto, Shuntaro Yoshida, Hiroyuki Isayama, Tomonori Yamada, Takeaki Matsuzawa, Toshiyuki Enomoto, Rika Kyo, Toshio Kuwai, Nobuto Hirata, Mamoru Shimada, Tomio Hirakawa, Koichi Koizumi, Yoshihisa Saida

**Affiliations:** 1 Department of Surgery, Kishiwada Tokushukai Hospital, 4-27-1 Kamori-cho, Kishiwada-shi, Osaka 596-8522 Japan; 2 Division of Surgery, Gastrointestinal Center, Yokohama Shin-Midori General Hospital, Yokohama, Japan; 3 Department of Endoscopy and Endoscopic Surgery, Graduate School of Medicine, The University of Tokyo, Tokyo, Japan; 4 Department of Gastroenterology, Graduate School of Medicine, The University of Tokyo, Tokyo, Japan; 5 Department of Gastroenterology, Graduate School of Medicine, Juntendo University, Tokyo, Japan; 6 Department of Gastroenterology, Japanese Red Cross Nagoya Daini Hospital, Nagoya, Japan; 7 Department of Digestive and General Surgery, Uonuma Institute of Community Medicine, Niigata University Medical and Dental Hospital, Niigata, Japan; 8 Department of Surgery, Toho University Ohashi Medical Center, Tokyo, Japan; 9 Department of Gastroenterology, Saiseikai Yokohamashi-Nanbu Hospital, Yokohama, Japan; 10 Department of Gastroenterology, National Hospital Organization Kure Medical Center and Chugoku Cancer Center, Kure, Japan; 11 Department of Gastroenterology, Kameda Medical Center, Kamogawa, Chiba Japan; 12 Department of Surgery, Toyonaka Midorigaoka Hospital, Osaka, Japan; 13 Department of Gastroenterology, Toyonaka Midorigaoka Hospital, Osaka, Japan; 14 Department of Gastroenterology, Tokyo Metropolitan Cancer and Infectious Disease Center Komagome Hospital, Tokyo, Japan

**Keywords:** Colon and rectal cancer, Intestinal obstruction, Self-expandable metallic stent, Bridge to surgery, Multicenter study, Prospective cohort study

## Abstract

**Background:**

Self-expandable metallic stenting (SEMS) for malignant colorectal obstruction (MCO) as a bridge to elective surgery (BTS) is a widely used procedure. The aim of this study was to assess short-term outcomes of SEMS for MCO as BTS.

**Methods:**

This study analyzed pooled data from BTS patients who were enrolled in two multicenter prospective single-arm observational clinical studies that used different stent types. Both studies were conducted by the Japan Colonic Stent Safe Procedure Research Group (JCSSPRG). The first study evaluated the WallFlex™ colonic stent for BTS or palliative treatment (PAL) from May 2012 to October 2013 and the second evaluated the Niti-S™ colonic stent from October 2013 to May 2014. Fifty-three facilities in Japan participated in the studies. Before each study started, the procedure had been shared with the participating institutions by posting details of the standard methods of SEMS placement on the JCSSPRG website. Patients were followed until discharged after surgery.

**Results:**

A total of 723 consecutive patients were enrolled in the two studies. After excluding nine patients, the remaining 714 patients were evaluated as a per-protocol cohort. SEMS placement was performed in 426 patients (312 WallFlex and 114 Niti-S) as BTS and in 288 as PAL. In the 426 BTS patients, the technical success rate was 98.1% (418/426). The clinical success rate was 93.8% (392/418). SEMS-related preoperative complications occurred in 8.5% of patients (36/426), perforations in 1.9% (8/426), and stent migration in 1.2% (5/426). Primary anastomosis was possible in 91.8% of patients (391/426), 3.8% of whom (15/393) had anastomosis leakage. The overall stoma creation rate was 10.6% (45/426). The postoperative complication rate was 16.9% (72/426) and mortality rate was 0.5% (2/426).

**Conclusions:**

SEMS placement for MCO as BTS is safe and effective with respect to peri-procedural outcomes. Further investigations are needed to confirm long-term oncological outcomes.

**Electronic supplementary material:**

The online version of this article (10.1007/s00464-018-6324-8) contains supplementary material, which is available to authorized users.

Malignant colorectal obstruction (MCO) is estimated to occur in 8–13% of colorectal cancer (CRC) cases and is the main reason for emergency surgery in patients with CRC [1–3]. Emergency surgery is associated with increased mortality and morbidity as well as a high chance of stoma creation, which reduces quality of life [4].

In efforts to avoid emergency surgery, self-expandable metallic stents (SEMS) were introduced for palliation of MCO in the 1990s [5, 6] and started being used as a bridge to elective surgery (BTS) in the same decade [7, 8]. In Japan, colonic stenting only become available in community hospitals including tertiary medical care centers in January 2012, when the procedure became covered by the national health insurance system. Until then, patients with left-sided CRC underwent transanal decompression tube placement to avoid emergency surgery. Although the decompression effect of transanal decompression tube placement is equivalent to that of colonic stenting, it causes greater discomfort for patients [9].

Colonic stenting can help to avoid emergency surgery. It also allows for a more thorough and detailed preoperative evaluation, including total colonoscopy, which can help to identify coexisting lesions and stage cancer more accurately. Patients can also resume oral intake and subsequently undergo elective surgery [10]. However, stenting may cause fatal complications such as perforation [11]. If perforation occurs, the risks of recurrent peritoneal carcinomatosis and death due to sepsis increase. Therefore, safe placement of the SEMS, without perforation, is vital.

In light of this, the Japan Colonic Stent Safe Procedure Research Group (JCSSPRG), affiliated with the Japan Gastroenterological Endoscopy Society, was set up in May 2012 to establish a safe procedure for colonic stenting for MCO. JCSSPRG conducted two multicenter prospective feasibility studies to investigate the safety and effectiveness of colonic stenting. The first study was conducted using only the WallFlex™ enteral colonic stent (Boston Scientific Corporation, Marlborough, MA) [12, 13], and the second subsequent study was conducted using only the Niti-S™ type D enteral colonic stent (TaeWoong, Inc., Seoul, South Korea) in the same setting.

This study used pooled analysis of the two studies to evaluate the short-term safety and effectiveness of SEMS placement as BTS in patients with MCO.

## Materials and methods

### Study design

The two multicenter prospective feasibility studies conducted by JCSSPRG were the WallFlex study (May 2012–October 2013) and the subsequent Niti-S study (October 2013–May 2014), involving a total of 53 facilities in Japan (14 academic centers and 39 community hospitals). Prior to the start of each study, institutional review board approval was obtained. The studies were registered in the Japan University Hospital Medical Information Network—Clinical Trials Registry (WallFlex study: UMIN000007953; Niti-S study: UMIN000011304).

In the WallFlex study, 312 of 518 consecutive patients enrolled in the registry were registered for SEMS placement as BTS [12, 13]. When enrollment was completed for the WallFlex study, the Niti-S study commenced using the same registry platform. In the Niti-S study, 112 of 205 consecutive patients enrolled in the registry were registered for SEMS placement as BTS. A total of 426 patients were registered for BTS in the two studies. Each patient was registered via the internet before or immediately after stent placement. All clinical data were collected prospectively. Patients scheduled for surgical resection were classified as “BTS” and patients not scheduled for surgical resection were classified as “palliative” (PAL). The present study analyzed data from the BTS group only.

To disseminate details about the SEMS procedure among the participating facilities before commencing each study, JCSSPRG launched a study group website (http://colon-stent.com/), posted the standard procedure as Mini-Guidelines (brief guidelines for safe placement of colonic stents), and held workshops to discuss a safe procedure for stent placement. The website was subsequently updated with the workshop content. The protocol of each study stated that participants must follow the Mini-Guidelines, and this was repeatedly mentioned at the workshops. A video of each stent placement procedure was also uploaded to the website, accompanied by a written explanation because the characteristics of each stent are quite different.

### Inclusion and exclusion criteria

The registry included patients with colorectal obstruction caused by malignant CRC or extracolonic cancer that required decompression as BTS or palliative care. Only patients with no previous colonic stenting were included in the registry. Exclusion criteria were enteral ischemia, suspected or impending perforation, intra-abdominal abscess/perforation, any contraindication to endoscopic treatment, and any use of the stent other than outlined specifically under the indications for use.

### Evaluation of obstructive symptoms

To assess oral intake level and abdominal symptoms before and after the procedure, JCSSPRG constructed a scoring system similar to that used to assess eating in patients with malignant gastric outlet obstruction [14]. The ColoRectal Obstruction Scoring System (CROSS) is described in detail elsewhere [12, 13]. Briefly, the patient’s oral intake level is assessed as follows: CROSS 0, requiring continuous decompression; CROSS 1, no oral intake; CROSS 2, liquid or enteral nutrient intake; CROSS 3, soft solids, low-residue, and full diet with symptoms of stricture; or CROSS 4, soft solids, low-residue, and full diet without symptoms of stricture (Table 1).

**Table 1:** The ColoRectal obstruction scoring system (CROSS)

Level of oral intake	Score
Requiring continuous decompression	0
No oral intake	1
Liquid or enteral nutrient intake	2
Soft solids, low-residue, and full diet with symptoms of stricture^a^	3
Soft solids, low-residue, and full diet without symptoms of stricture	4

^a^Symptoms of stricture include abdominal pain/cramps, abdominal distention, nausea, vomiting, constipation, and diarrhea and are related to gastrointestinal transit

### Stent device and procedure

The WallFlex™ enteral colonic stent (Boston Scientific Corporation) used in the Wallflex study was uncovered. The stent placed was 6, 9, or 12 cm in length, with flare flange/mid-body diameters of either 27/22 or 30/25 mm.

The Niti-S™ type D enteral colonic stent (TaeWoong Medical Co., Ltd.) used in the Niti-S study was uncovered and had no flare flange. The stent placed was 6, 8, 10, or 12 cm in length and 18 or 22 mm in diameter.

The stent placement procedure was performed under fluoroscopic and endoscopic guidance, in accordance with the Mini-Guidelines published on the JCSSPRG website. Briefly, (1) to prevent the visual field from deteriorating due to bleeding, biopsy should be kept to a minimum. (2) The guidewire should always be used first to break through the stenosis in through-the-scope stent placement. (3) It is recommended that the tumor be marked with an endoscopic metal clip to identify and visualize the anal side margin. (4) No dilatation of the stricture by balloon or bougie is allowed.

### Outcome measures

In this study, data were analyzed for BTS patients only (i.e., those scheduled for elective surgery after stent placement). The follow-up period was time to discharge after surgery. Technical success of BTS was defined as accurate SEMS placement with adequate stricture coverage on the first attempt with no adverse events. Clinical success of BTS was defined as decompression and relief of obstructive symptoms until surgery with no stent-related complications or need for endoscopic re-intervention or emergency surgery [13].

Procedure-related adverse events were defined as perforation, re-obstruction, stent migration, infection/fever, abdominal pain, and tenesmus. Silent perforation (i.e., exposure of the stent outside of the bowel at the time of surgery with no symptoms after stent placement) was not considered a complication because the patients had no peritonitis, localized inflammation, or abdominal pain that affected the clinical course, thus making it difficult for the physicians to recognize it as a complication.

Procedure-related complications and adverse events were examined separately for early complications up to 7 days after SEMS placement and for late complications after day 8.

Operative outcomes were evaluated based on whether the elective operation could be performed on schedule or whether stoma creation was required. Surgical complications and length of postoperative hospital stay were also examined.

### Statistical analysis

Continuous variables are expressed as medians and range or interquartile range, as appropriate. Continuous and nominal variables were compared using the χ^2^ test.

## Results

**Fig. 1:**
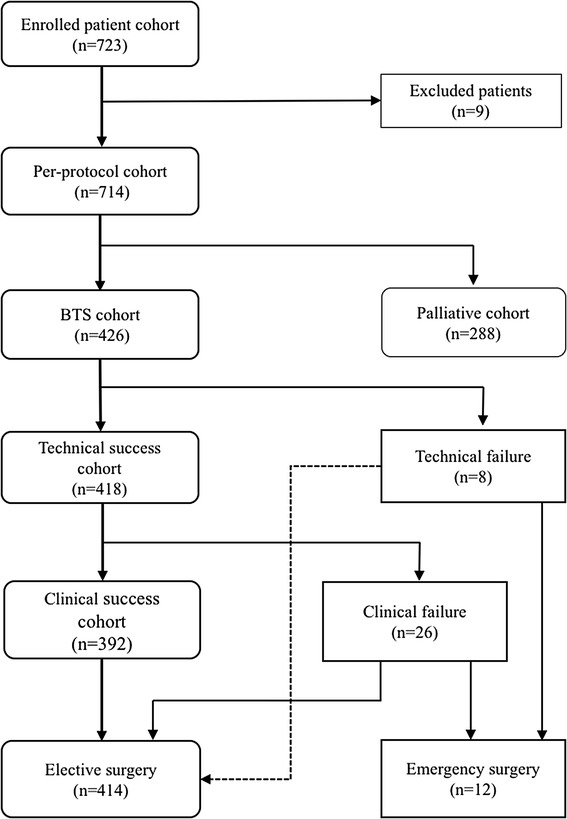
Patient analysis flowchart. *BTS* bridge to surgery

A flowchart of patient allocation is shown in Fig. 1. Of 723 consecutive patients enrolled, nine were excluded because of mild stenosis identified on colonoscopy (*n* = 5), gastrocolic fistula (*n* = 1), deterioration of respiratory status (*n* = 1), use of another type of stent (*n* = 1), and adhesive small bowel obstruction (*n* = 1). The remaining 714 patients were evaluated as a per-protocol cohort. Colonic stenting was performed as BTS for malignant colorectal obstruction in 426 patients (312 WallFlex and 114 Niti-S) and as PAL in 288 patients. Pooled data of the BTS patients were analyzed to determine the short-term outcomes of SEMS as BTS. All BTS patients could be followed up until they were discharged after surgery.

### Baseline characteristics

Of the 426 BTS patients, 239 (56.1%) were men and 187 (43.9%) were women. Mean age was 69.4 years (Table 2). Primary CRC was reported in 419 patients (98.4%). Four of the remaining seven patients had locally recurrent CRC (*n* = 2) or extracolonic cancer (*n* = 2). Although three patients had benign stricture, they were not excluded from the analysis because they had a clinical diagnosis of CRC based on contrast-enhanced computed tomography of the abdomen and colonoscopy findings. SEMS placement and surgical resection was then performed as for malignancy. Pathological examination after surgery identified the strictures as benign lesions (two were simple ulcers and one was endometriosis).

**Table 2:** Baseline patient demographic and tumor characteristics in the patients of the BTS cohort

Patient characteristics (*n* = 426)	
Age (years) (mean ± SD)	69.4 ± 11.7
Sex [% (*n*)]	
Male	56.1 (239)
Female	43.9 (187)
Any stricture-related symptoms [% (*n*)]	94.8 (404)
No symptom	5.2 (22)
CROSS before stent placement [% (*n*)]	
0	36.4 (155)
1	30.0 (128)
2	13.1 (56)
3	12.9 (55)
4	7.5 (32)
Etiology of colorectal stenosis [% (*n*)]	
Primary colorectal cancer	98.4 (419)
Localized (*n* = 419)	74.5 (312)
With distant metastasis (*n* = 419)	26.7 (112)
Locally recurrent colorectal cancer	0.5 (2)
Other extrinsic cancer	0.5 (2)
Benign lesion	0.7 (3)
Location of the tumor [% (*n*)]	
Right-sided colon	23.2 (99)
Left-sided colon	73.7 (314)
Rectum	3.1 (13)
UICC-TNM classification for primary CRC patients (*n* = 419) [% (*n*)]	
1	0.5 (2)
2	34.6 (145)
3	37.7 (158)
4	27.2 (114)

*BTS* bridge to surgery, *CROSS* ColoRectal obstruction scoring system, *UICC* Union for International Cancer Control

The tumor was located proximal to the splenic flexure in 23.2% of patients, in the left colon in 73.7%, and in the rectum in 3.1%. At preoperative staging, 74.5% of the patients had localized CRC without metastatic disease and 26.7% of the patients had distant metastasis. Among the 419 patients with primary CRC, the postoperative UICC-TNM stage (7th edition) of primary CRC was stage I in 2 patients (0.5%), stage II in 145 patients (34.6%), stage III 158 patients (37.7%), and stage IV in 114 patients (27.2%; Table 2).

Symptoms of acute colonic obstruction (e.g., worsening pattern of defecation, abdominal pain/cramps, bloating, and nausea/vomiting) were identified in 404 patients (94.7%); the remaining 22 patients (5.2%) had no stricture-related symptoms. Oral intake was assessed as CROSS 0 in 155 patients (36.4%), CROSS 1 in 128 patients (30.0%), CROSS 2 in 56 patients (13.1%), CROSS 3 in 55 patients (12.9%), and CROSS 4 in 32 patients (7.5%; Table 2).

### Technical success

The technical success rate was 98.1% (418/426 patients; Table 3). Stent placement was unsuccessful because of inability to pass the guidewire through the tumor stricture (*n* = 3), perforation by the guidewire (*n* = 3), inability to visualize the tumor endoscopically (*n* = 1), and migration to the proximal side (*n* = 1). Migration to the proximal side actually occurred in two patients; however, according to the definition of accurate SEMS placement on the first attempt, one case was regarded as a technical success because stent re-placement was immediately successful; the other case was regarded as a technical failure because stent re-placement was tried on another occasion. Of the eight technical failures, four patients underwent emergency surgery (two Hartmann’s resections and two palliative colostomies) and four patients received elective surgery but needed preoperative fasting (except for clear fluids) until surgery (one Hartmann’s resection and three primary anastomosis operations). Seven of the technical failures occurred in the WallFlex study (technical success rate, 97.8%); the single technical failure in the Niti-S study (technical success rate, 99.1%) was due to migration to the proximal side.

**Table 3:** Technical success rate and the cause of failure

	WallFlex study (*n* = 312)	Niti-S study (*n* = 114)	Total (*n* = 426)
Technical success rate [% (*n*)]	97.8 (305)	99.1 (113)	98.1 (418)
Cause of failure [% (*n*)]			
Inability to pass the guidewire	1.0 (3)	0 (0)	0.7 (3)
Perforation by the guidewire	1.0 (3)	0 (0)	0.7 (3)
Inability to endoscopically visualize the tumor	0.3 (1)	0 (0)	0.2 (1)
SEMS migration to the proximal colon	0 (0)	0.9 (1)	0.2 (1)

*SEMS* self-expandable metallic stent

### Clinical success

Clinical success for BTS was achieved in 392 patients (Table 4). When the eight patients with technical failure were excluded in order to evaluate the clinical effect of SEMS, the clinical success rate was 93.8% (392/418). In the per-protocol cohort, it was 92.0% (392/426).

**Table 4:** Clinical success rate and adverse events

	WallFlex study	Niti-S study	Total
	*n* = 305	*n* = 113	*n* = 418
Clinical success rate after technical success [% (*n*)]	92.1 (281)	98.2 (111)	93.8 (392)

Adverse events causing clinical failure in technical success cohort (*n* = 418)	*n* = 305	*n* = 113	*n* = 418	Early times (within 7 days)	Late times (after 7 days)
Perforation [% (*n*)]	1.6 (5)	0 (0)	1.2 (5)	4	1 (19 days)
Stent migration [% (*n*)]	1.3 (4)	0 (0)	1.0 (4)	3	1 (28 days)
Sepsis [% (*n*)]	0.3 (1)	0 (0)	0.2 (1)	1	0
Obstructive colitis [% (*n*)]	0.6 (2)	0 (0)	0.5 (2)	2	0
Persistent obstruction [% (*n*)]	1.0 (3)	0.9 (1)	0.9 (4)	4	0
Re-obstruction/stool impaction [% (*n*)]	0.3 (1)	0.9 (1)	0.5 (2)	1	1 (15 days)
Fever [% (*n*)]	1.3 (4)	0 (0)	0.9 (4)	1	3 (11 days, 22 days, 24 days)
Tenesmus/abdominal pain [% (*n*)]	1.0 (3)	0 (0)	0.7 (3)	3	0
Acute appendicitis [% (*n*)]	0.3 (1)	0 (0)	0.2 (1)	1	0
Others [% (*n*)]	0 (0)	1.8 (2)	0.5 (2)	2	0
Silent perforation [% (*n*)]	1.3 (4)	0 (0)	1.0 (4)		

Adverse events in per-protocol cohort (*n* = 426)	*n* = 312	*n* = 114	*n* = 426
Total perforation rate including technical failure [% (*n*)]	2.6 (8)	0 (0)	1.9 (8)
Total perforation rate including technical failure and silent perforation [% (*n*)]	3.8 (12)	0 (0)	2.8 (12)
Total stent migration rate including technical failure [% (*n*)]	1.3 (4)	0.9 (1)	1.2 (5)
Overall complication rate including technical failure [% (*n*)]	9.9 (31)	4.4 (5)	8.5 (36)

Regarding stent-related complications, perforation after technical success occurred in five patients: perforation from contact with the SEMS (*n* = 2), perforation of the proximal side of the colon not contacting the tumor (*n* = 1), perforation by the tumor itself (*n* = 1), and perforation from acute appendicitis (*n* = 1). In the two cases of perforation from contact with the SEMS, perforation occurred at the site where the edge of the stent was in contact with the intestinal wall; emergency surgery was performed on day 5 in one case and on day 19 in the other.

A total of eight patients, including the above five patients with perforation, underwent emergency surgery after technical success. The other three cases were due to obstructive colitis (*n* = 2) or sepsis caused by necrotizing colitis (*n* = 1). In these eight cases of emergency surgery, four patients underwent Hartmann surgery and four underwent primary anastomosis.

Silent perforation occurred in four patients, all in the WallFlex study. The total perforation rate was 1.9% when including procedure-related perforation and SEMS or tumor-related perforation but not silent perforation, and was 2.8% when including silent perforation. No perforations, including silent perforation, were reported in the Niti-S study.

Other adverse events were migration in five patients (1.2%), persistent obstruction in 4 (0.9%), fever in 4 (0.9%), abdominal pain/tenesmus in 3 (0.7%), and stool impaction in 2 (0.5%). In total, five patients had complications with the Niti-S™ stent, all of which were early complications (within 7 days). The overall complication rate including technical failure was 8.5%.

The top ten enrolling facilities registered half of the patients, but they accounted for only one-third of the morbidity rate. There were fewer complications in the more experienced facilities, with no significant difference between academic hospitals and community hospitals, because the number of patients registered was very low in some academic hospitals (Supplemental Table S1).

### SEMS profile

In the WallFlex study, the most common length was 6 cm, used in 200 patients (65.6%). In the Niti-S Study, the most common length was 8 cm, used in 45 patients (39.5%). In both studies, most patients were treated using a stent with a mid-body diameter of 22 mm (381/419, 90.9%); 280 patients in the WallFlex study and in 102 patients in the Niti-S study. There were 26 cases of clinical failure after technical success, but the profiles of stent length and diameter used were not different from those used in the cases of clinical success (Supplemental Table S2).

### Surgery after BTS colonic stenting

After the technical success of SEMS placement, 410 patients underwent elective surgery. Median time from SEMS placement to elective surgery was 17 days. The tumor was resectable in 97.6% of cases. Open surgery was performed in 38.8% of cases and laparoscopic surgery in 61.2%, and the conversion rate was 10.0%. Primary anastomosis without a diverting stoma was performed in 382 patients (89.7%) in the per-protocol cohort. Three of them required emergency re-operation due to anastomotic leakage and needed a diverting stoma. Stoma creation was also required in the following cases in the per-protocol cohort: nine involving primary anastomosis with a diverting stoma, 24 involving Hartmann’s resection, and nine involving palliative colostomy. Palliative bypass surgery was performed in two patients. The overall stoma creation rate was 10.6% (Table 5).

**Table 5:** Surgery after BTS colonic stenting

Elective surgery after technical success (*n* = 410)	
Open surgery [% (*n*)]	38.8 (159)
Laparoscopic surgery [% (*n*)]	61.2 (251)
Conversion [% (*n*)] (*n* = 251)	10.0 (25)
The median time from SEMS placement to elective surgery, days (interquartile range) (*n* = 410)	17 days (12–25)
Tumor resectable [% (*n*)]	97.6 (400)
Operative procedure in per-protocol cohort (*n* = 426)	
Primary anastomosis [% (*n*)]	91.8 (391)
Without diverting stoma [% (*n*)]	89.7 (382)
With diverting stoma [% (*n*)]	2.1 (9)
Hartmann [% (*n*)]	5.6 (24)
Palliative colostomy only [% (*n*)]	2.1 (9)
Palliative bypass [% (*n*)]	0.5 (2)
Re-operation with diverting stoma for anastomotic leakage [% (*n*)]	0.7 (3)
Overall stoma creation rate [% (*n*)] (*n* = 426)	10.6 (45)

The overall morbidity rate was 16.9%. Anastomotic leakage occurred in 15 of 393 patients (3.8%), 391 who had a primary anastomosis and two who underwent palliative bypass. Twelve of these 15 patients recovered with conservative management and the remaining three required re-operation with diverting stoma creation. Other complications were 22 cases of wound infection (5.2%), 21 of bowel obstruction (4.9%), six of intraperitoneal abscess (1.4%), three of pulmonary complications (0.7%), two each of sepsis and renal failure (0.5%), and one case of deep venous thrombosis (0.2%). Median postoperative hospital stay was 14 days. Postoperative mortality was seen in two patients, for an overall mortality rate of 0.5%. The cause of death in both patients was cancer progression (Table 6).

**Table 6:** Morbidity and mortality rate and postoperative hospital stay

	*n* = 426
Total postoperative complications [% (*n*)]	16.9 (72)
Anastomotic leakage [% (*n*)], (*n* = 393)	3.8 (15/393)
Conservative treatment [% (n)], (*n* = 393)	3.1 (12/393)
Emergency re-operation with stoma [% (*n*)], (*n* = 393)	0.8 (3/393)
Wound infection [% (*n*)]	5.2 (22)
Bowel obstruction [% (*n*)]	4.9 (21)
Intraperitoneal abscess [% (*n*)]	1.4 (6)
Pulmonary complication [% (*n*)]	0.7 (3)
Sepsis [% (*n*)]	0.5 (2)
Renal failure [% (*n*)]	0.5 (2)
Deep vein thrombosis [% (*n*)]	0.2 (1)
Postoperative mortality rate [% (*n*)]	0.5 (2)
Postoperative hospital stay; median (interquartile range)	14 days (10–22)

## Discussion

JCSSPRG conducted two prospective multicenter series to evaluate the effectiveness and safety of colonic stenting with two types of SEMS. The total number of cases in the two studies was 723. While this is a considerably large number, the complication rate was relatively low compared with previous reports [15]. For the 513 patients in the initial WallFlex study [12, 13] and the 201 patients in the subsequent Niti-S study, the respective technical success rates at 7 days were 97.9 and 98.0%, the clinical success rates were 95.5 and 96.5%, and the perforation rates were 2.1 and 0%, representing fairly good outcomes.

In the last decade, eight systematic reviews based on randomized controlled trials (RCT)s have been published that compared patients who underwent elective surgery after colon stenting as BTS with those who underwent emergency surgery [16–23]. One of these, by Cennamo et al., was based on eight RCTs and reported technical and clinical success rates of 73.5 and 72%, respectively [17]; the technical and clinical success rates reported in the present study are well above these. The total complication rate and perforation rate for stent placement have been reported to be 10 and 8.4%, respectively, and our rates of 8.5 and 1.9% are well below these. Our results are also consistent with those reported by the WallFlex Colonic Registry Group (technical success rate, 98%; clinical success rate, 94%), which was a large multicenter prospective observational study conducted prior to our study [24, 25].

In general, the more advanced the obstruction is, the more difficult it is to confirm the lumen of the stricture portion, and therefore the procedure becomes more technically demanding in cases of severe obstruction. Consequently, the complication rate is likely to increase [26]. In our study, patients with CROSS 3 or 4, who have relatively less advanced obstruction, accounted for 20.4% of the subjects, and their inclusion may have influenced the study results.

This study was a pooled analysis of the initial WallFlex study and the subsequent Niti-S study. Outcomes were more favorable in the latter study. In particular, the clinical success rate was significantly better and the perforation rate was 0%. A major contributing factor is likely to be the improvement in operators’ skills between the two studies. In Japan, before colonic stenting was covered by the national health insurance system in January 2012, it was performed in only a few academic centers engaging in clinical trials. Given that 39 of the 53 facilities involved in the WallFlex and Niti-S studies were community hospitals, most of the participants probably had limited experience in stent placement before the WallFlex study started. Most of the participating operators were involved in both studies, which were conducted in the same settings, so their skills were likely improved in the second study [27, 28].

Other factors could have compensated for the lack of technical experience among participants in the early stages but still led to the favorable results obtained in the Wallflex study. JCSSPRG posted Mini-Guidelines outlining the standard procedure for SEMS placement on the study group website. In addition, the study group held workshops to examine the procedural details and share techniques, including the selection of guidewires and endoscopes, the necessity of an internal marker, and technical tips on how to break through stricture with the guidewire. The Mini-Guidelines were then updated based on these details. Thus, the participants had a number of opportunities to become familiar with the technique before performing it, which may have helped with the favorable results of the first study. They also then had ready access to similar information in the subsequent Niti-S study.

Certain differences in the characteristics of the WallFlex™ and Niti-S™ stents may also have influenced the results. Compared with WallFlex™, which has a comparatively strong straightening force, Niti-S™ has the ability to adapt to curves and remain in a bent shape, and this may have been advantageous [29, 30]. In fact, two cases of perforation caused directly by the stent occurred in the WallFlex study, when the stent margin hit the intestinal wall and caused perforation; emergency surgery was required in both cases. In addition, silent perforation, which may be more likely to occur with a stent that has strong straightening force, was reported in four WallFlex cases. No silent perforation was reported in the Niti-S study.

The diameter and length of a stent may be related to stent complications, especially perforation and migration [31]. Theoretically, large-diameter SEMS can increase the risk of stent-related perforation because the stent will expand the tumor more considerably. A study conducted by Hooft et al. involved the use of SEMS that were 25 mm in diameter, but the study was closed prematurely due to a high perforation rate [32]. The colonic stenting guidelines of the European Society of Gastrointestinal Endoscopy (ESGE), which were released in 2014 [33], state that stents ≤ 24 mm in diameter are not recommended because of increased risk of complications such as migration. Geraghty et al. reported no significant difference between the clinical success rate of stents ≥ 25 and < 25 mm in diameter (95.2% vs. 87.4% p = 0.161) [34]. In the present study, 22-mm stents were the most commonly used stents in both the WallFlex study (92%) and Niti-S study (90%). There was no difference in clinical success rate between this diameter of 22 mm and other diameters. Stent migration rate in the present study was 1.2%, which is acceptable. The perforation rate of 1.9% is also acceptable and is lower than that reported in other studies. The stent diameter of 22 mm may be optimal to reduce the incidence of these complications.

Stent length was not identified as a risk factor in previous reports [11, 35]. Stent- or tumor-induced perforation in the present study occurred in five patients in the WallFlex study only, where stent length was 6 cm in three cases and 9 cm in two cases. Therefore, complication rate and stent length do not appear to be related in this study. However, because perforation from contact of the stent edge with the intestinal wall occurred in two patients, it may be better for the length that protrudes from both sides of the tumor edge to be as short as possible, especially for WallFlex™ stents with their strong straightening force [33].

Long-term oncological outcomes should be monitored. The ESGE guidelines clearly state that colonic stenting as BTS is not recommended as standard treatment for symptomatic left-sided MCO [33]. Of the papers cited in the guidelines that support this recommendation, only studies with a high perforation rate reported a significant difference in oncological outcomes [36–38]. In long-term follow-up of the Stent-in-2 trial, the oncological outcomes were poor in the group in which perforation occurred after stenting, and long-term oncological outcomes did not differ between the group in which the stent could be placed without perforation and the emergency surgery group [38]. Stenting might not worsen long-term oncological outcomes if the stent can be placed with a low risk of perforation, as in the present study. Indeed, in a relatively large RCT that was published recently, there were no differences in oncological outcomes with respect to overall survival (OS) and disease-free survival (DFS) between the two groups, although the follow-up period of 3 years was quite short [39]. JCSSPRG plans to start an RCT to compare a group undergoing early surgery after fasting and a group undergoing elective surgery after colonic stenting in patients with CROSS 1 or 2. If a low perforation rate were to be observed, similar to that in the present study, it may mean that stent placement does not adversely impact oncological outcomes.

This study has three limitations. First, this was a non-randomized, single-arm study and it was not possible to compare the stent group with the emergency surgery group. Second, subjects included many asymptomatic patients (5.2%) and CROSS 3 or 4 patients (20.4%) who could eat at least a soft diet. It is undeniable that this affected the low perforation and complication rate. Third, long-term oncological outcomes were not evaluated. Patients enrolled in this study continue to be followed up and we intend to report on their long-term oncological outcomes in the future.

In conclusion, the analysis of pooled data from two large multicenter prospective feasibility studies of 426 MCO patients showed that SEMS placement for MCO as BTS is safe and effective with respect to short-term peri-procedural outcomes, and that subsequent elective surgery can be performed with low morbidity, low mortality, and a low stoma creation rate. Further investigations are needed to confirm the long-term oncological outcomes of SEMS placement for MCO as BTS.

### Electronic supplementary material

Below is the link to the electronic supplementary material.

Supplementary material 1 (DOCX 48 KB)

Supplementary material 2 (DOCX 67 KB)

## Appendix

The Japan Colonic Stent Safe Procedure Research Group includes the following members in addition to the authors: Tatsuya Osuga, Aijinkai Takatsuki General Hospital, Takatsuki, Japan; Hiroo Matsushita, Akita Red Cross Hospital, Akita, Japan; Mitsuru Goto, Asahikawa Kosei Hospital, Asahikawa, Japan; Shungo Endo, Fukushima Medical University Aizu Medical Center, Aizu-Wakamatsu, Japan; Yohei Kurose, Fukuyama City Hospital, Fukuyama, Japan; Shigeru Yamagishi, Fujisawa City Hospital, Fujisawa, Japan; Katsuya Ota, Higashiosaka City General Hospital, Higashiosaka, Japan; Masanori Yoshimitsu, Hiroshima City Asa Hospital, Hiroshima, Japan; Satoshi Ikeda, Hiroshima Prefectural Hospital, Hiroshima, Japan; Rintaro Moroi, Iwate Prefectural Isawa Hospital, Oshu, Japan; Routa Noaki, Kawaguchi Municipal Medical Center, Kawaguchi, Japan; Hirofumi Kawamoto, Kawasaki Medical School, Okayama, Japan; Hirotoshi Hasegawa, Keio University School of Medicine, Tokyo, Japan; Atsushi Yamauchi, Kitano Hospital, Osaka, Japan; Fuminori Teraishi, Kochi Health Sciences Center, Kochi, Japan; Kohei Takayasu, Kyorin University Hospital, Mitaka, Japan; Takahiro Horimatsu, Kyoto University Hospital, Kyoto, Japan; Yoshinori Kushiyama, Matsue Red Cross Hospital, Matsue, Japan; Hiroaki Naota, Matsusaka Chuo General Hospital, Matsusaka, Japan; Takuya Yamaguchi, Mimihara General Hospital, Sakai, Japan; Shigenori Masaki, Miyanomori Memorial Hospital, Sapporo, Japan; Taku Sakamoto, National Cancer Center Hospital, Tokyo, Japan; Chizu Yokoi, National Center for Global Health and Medicine, Tokyo, Japan; Masafumi Inomata, Oita University Faculty of Medicine, Oita, Japan; Nobuya Obana, Osaki Citizen Hospital, Osaki, Japan; Masayoshi Horimoto, Osaka Saiseikai Senri Hospital, Suita, Japan; Shinei Kudo, Showa University Northern Yokohama Hospital, Yokohama, Japan; Yasushi Nakamura, Takano Hospital, Kumamoto, Japan; Mitsunori Ushigome, Toho University Omori Medical Center, Tokyo, Japan; Takeshi Ohki, Tokyo Women’s Medical University, Tokyo, Japan; Hiroyuki Kato, Tokyo Women’s Medical University Medical Center East, Tokyo, Japan; Shiro Hayashi, Toyonaka Municipal Hospital, Toyonaka, Japan; Kensuke Kubota, Yokohama City University Hospital, Yokohama, Japan; Kunihiko Amano, Saitama Medical Center, Saitama Medical University, Moroyama, Japan; Masanori Yoshino, Nippon Medical School Musashi Kosugi Hospital, Kawasaki, Japan; Takuji Kawamura, Japanese Red Cross Kyoto Daini Hospital, Kyoto, Japan; Yorinobu Sumida, Kushu Medical Center, Fukuoka, Japan; Noriko Watanabe, Mie Chuo Medical Center, Tsu, Japan; Hideto Egashira, Shonan-kamakura General Hospital, Kamakura, Japan; Takahisa Kayahara, Kurashiki Central Hospital, Kurashiki, Japan; Hideki Kanazawa, Sagamihara National Hospital, Sagamihara, Japan; Michiaki Watanabe, Atsugi City Hospital, Atsugi, Japan; and Kensuke Kubota, Yokohama City University Hospital, Yokohama, Japan.
