# Supplementary material for: Self-expandable metallic stenting as a bridge to surgery for malignant colorectal obstruction: pooled analysis of 426 patients from two prospective multicenter series
Source: Surg Endosc. 2018 Jul 13;33(2):499–509. doi: 10.1007/s00464-018-6324-8 (PMC6342866; doi:10.1007/s00464-018-6324-8)
Supplement: Supplementary file 1 — Supplementary material 1 (DOCX 48 KB) [file 464_2018_6324_MOESM1_ESM.docx]

Supplemental Table S1. Complication rate of stent complications by type of facility

|  | With any complication  (n = 36) | No complication  (n = 390) | (χ2 test) |
| --- | --- | --- | --- |
| Top 10 enrollment facilities  (226 patients), % (n) | 5.8 (13) | 94.2 (213) | p = 0.03 |
| Outside of top 10 facilities (200 patients), % (n) | 11.5 (23) | 88.5(177) |  |
|  |  |  |  |
| Academic centers  (14 hospitals/ 95 patients), % (n) | 9.5 (9) | 90.5 (86) | p = 0.68 |
| Community hospitals  (39 hospitals/331 patients), % (n) | 8.2 (27) | 91.8(304) |  |
