# Supplementary material for: Self-expandable metallic stenting as a bridge to surgery for malignant colorectal obstruction: pooled analysis of 426 patients from two prospective multicenter series
Source: Surg Endosc. 2018 Jul 13;33(2):499–509. doi: 10.1007/s00464-018-6324-8 (PMC6342866; doi:10.1007/s00464-018-6324-8)
Supplement: Supplementary file 2 — Supplementary material 2 (DOCX 67 KB) [file 464_2018_6324_MOESM2_ESM.docx]

Supplemental Table S2. Profiles of stent length and diameter

|  | Clinical success | Clinical failure | (χ2 test) | | Total |
| --- | --- | --- | --- | --- | --- |
| WallFlex (n = 305) | | | | | |
| Length, n (%) | n = 281 | n = 24 |  | | n = 305 |
| 6cm | 182 (64.8) | 18 (75.0) | p = 0.35 | | 200 (65.6) |
| 9cm | 94 (33.5) | 5 (20.8) |  |  | 99 (32.5) |
| 12cm | 5 (1.8) | 1 (4.2) |  |  | 6 (2.0) |
| Diameter, n (%) |  |  |  |  | |
| 22/27mm | 260 (92.5) | 20 (83.3) | p = 0.12 | | 280 (91.8) |
| 25/30mm | 21 (7.5) | 4 (16.7) |  |  | 25 (8.2) |
| Niti-S (n = 113) | | | | | |
| Length, n (%) | n = 111 | n = 2 |  | | n = 113 |
| 6cm | 30 (27.0) | 0 (0) | p = 0.69 | | 30 (26.5) |
| 8cm | 44 (39.6) | 1 (50.0) |  |  | 45 (39.8) |
| 10cm | 25 (22.5) | 1 (50.0) |  |  | 26 (23.0) |
| 12cm | 12 (10.8) | 0 (0) |  |  | 12 (10.6) |
| Diameter, n (%) |  |  |  |  | |
| 18mm | 12 (10.8) | 0 (0) | p = 0.62 | | 12 (10.6) |
| 22mm | 99 (89.2) | 2 (100) |  |  | 101 (89.4) |
